# Sequelae Following Radical Parotidectomy: The Role of the Reconstructive Surgeon

**Published:** 2014-03-31

**Authors:** James Allan, Jeon Cha, Johnny Kwei, John G. Vandervord

**Affiliations:** Burns, Plastic, Reconstructive and Maxillofacial Surgery, Royal North Shore Hospital, St Leonards, Sydney, Australia

**Keywords:** facial nerve, parotidectomy, nerve graft, regional muscle transfers, free tissue transfer

## DESCRIPTION

A 45-year-old man with a deep lesion to the parotid (shown to be a basal cell carcinoma on biopsy) underwent a radical parotidectomy following discussions in a multidisciplinary meeting. During the extirpation surgery, portions of the facial nerve were sacrificed following examination under frozen section.

## QUESTIONS

1. **What are primary goals of a plastic surgeon following disruption of the facial nerve during a surgical resection?**
2. **What nerve options are available for facial nerve reconstruction?**
3. **What are the local muscle options that are available for dynamic reconstruction following facial nerve disruption?**
4. **What are the other considerations that need to be addressed following a radical parotidectomy?**

## DISCUSSION

Disruption of the facial nerve is a sequelae of radical parotidectomy. It has a high prevalence in Australia due to the relative high incidence of metastatic squamous cell carcinoma to the parotid.^1^ Loss of the facial nerve may result in the loss of protection to the eyes and inspiratory nasal and oral competence. Effective and appropriate communication may also be affected with those unable to appropriately express emotion noting a deleterious effect on their psyche.^2^ It is not possible to effectively restore the intricate interplay between the mimetic muscles. Consequently, the primary goals must be to protect the crucial functions of vision, nasal airflow, and oral continence; to restore facial symmetry at rest; and to attempt to achieve symmetry during movement.^3^

**Figure 1:**
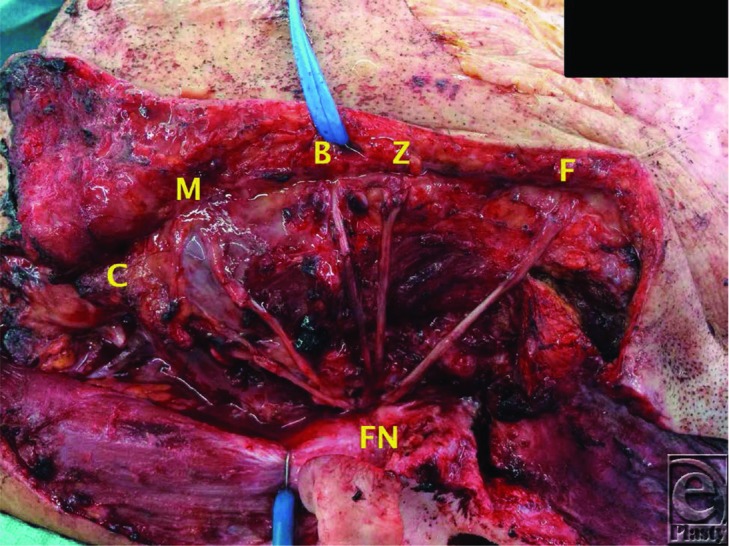
Sural nerve grafts following end-to-end neurorrhaphies to the frontal (F), zygomatic (Z), buccal (B), marginal mandibular (M), cervical (C) branches, and the facial nerve trunk (FN).

Where the facial nerve defect is small, primary repair achieves the best outcomes.^4^ In cases of radical parotidectomy, direct repair is often not possible due to the defect size. If the facial nerve trunk is preserved then cable grafting to the branches should be considered. Often the great auricular nerve is exposed during the extirpation surgery and concurrent neck dissection. It may be used as a donor graft due to its caliber, low donor site morbidity, and length. Other graft options include the dorsal cutaneous nerve of the foot and the sural nerve. The sural nerve in particular is useful due to the ease of access, caliber, its long length allowing cable grafting, and relatively low donor site morbidity with the loss of sensation to the lateral foot being tolerated well by most patients (Fig 1).^5^ Other options include nerve transfers using the hypoglossal nerve for either end-to-end or end-to-side neurorrhaphy. The nerve to masseter is another alternative for a nerve transfer, which has been shown to be effective in producing a dynamic and spontaneous response following activation.^6^

**Figure 2:**
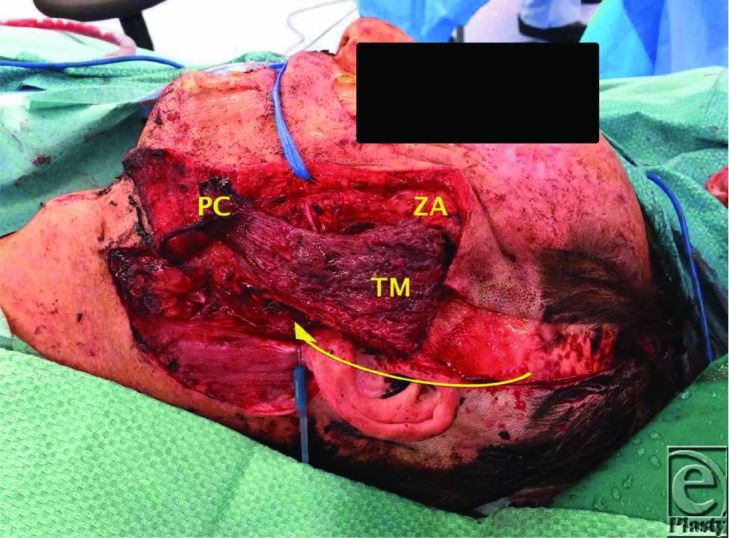
A turndown temporalis muscle flap (TM) utilizing the middle half only to minimize temporal hollowing with pericranial extensions (PC) (ZA = level of the zygomatic arch).

Static reconstruction refers to slings, which can be used to support the external nasal valve, maintain oral continence, and provide symmetry at rest. Dynamic reconstruction is a more desirable outcome for the patient and can be achieved with either regional or free muscle transfers. The masseter and temporalis are 2 regional muscle transfers that are particularly useful in improving oral function and facial communication. The detached end of either muscle can be attached to the oral commissure and the philtral region, whereupon clenching the teeth enables dynamic excursion of the lateral aspect of the mouth to approximate a smile.^6^ The temporalis muscle may be used anterograde or as a turndown flap. If used as a turndown flap, the total length is often inadequate and an extension of pericranium may be needed to allow inset of temporalis into the commissural region. The drawback of this procedure is the resultant temporal hollowing if the whole of the muscle is used. To minimize this, a partial or anterograde temporalis may be harvested (Fig 2). Loss of the marginal mandibular branch of the facial nerve will result in paralysis of the depressor labii inferioris, depressor anguli oris, and mentalis producing an asymmetrical smile with the lower lip appearing flat and inwardly rotated on the affected side. A local muscle transfer to correct this imbalance is the anterior belly of the digastric muscle, with the central tendon inserted into the lower lip vermillion border 1 to 2 cm medial to the oral commissure.^7^

**Figure 3:**
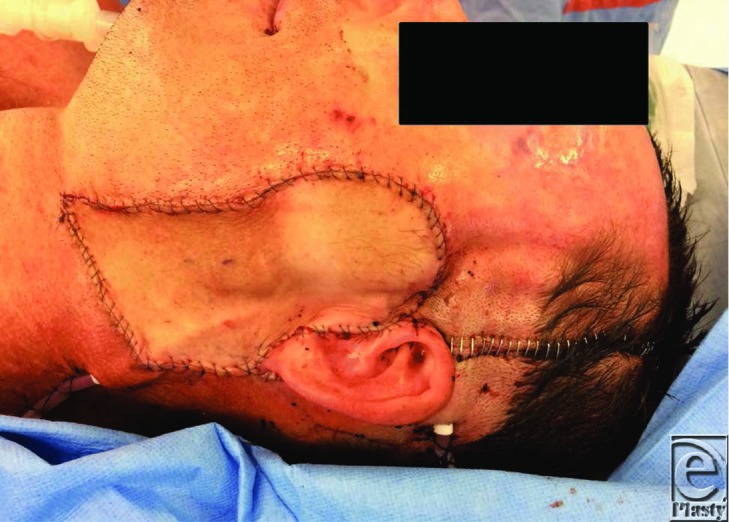
A radial forearm free flap was inset to improve the contour defect and to provide soft tissue coverage. A temporary tarsorrhaphy was sited on the left eyelids to provide ocular protection (not visible).

Additional concerns following radical parotidectomy include depressed contours in the parotid region and loss of cutaneous coverage.^3^ Local options that are available to address these concerns include a cervicofacial or submental flap. Free fasciocutaneous tissue transfer, however, provides the gold standard for contour restoration and skin coverage (Fig 3). Salivary fistulas and Frey's syndrome must also be considered as potential complications following a radical parotidectomy.^8^

The patient described in this case had the facial nerve excised to the level of the main trunk following frozen section biopsies. His potential deficits were corrected with a temporary tarsorrhaphy (to protect ocular function), a segmental temporalis turndown flap with pericranial extensions (for oral competence and dynamic motion). In addition, sural nerve cable grafts were used to bridge the defect between the facial nerve trunk and the 5 distal branches of the facial nerve. A radial forearm free flap was used to improve his contour defect and to provide cutaneous coverage.
